# The interaction effect of rs4077515 and rs17019602 increases the susceptibility to IgA nephropathy

**DOI:** 10.18632/oncotarget.20401

**Published:** 2017-08-23

**Authors:** Changwei Wu, Guisen Li, Li Wang

**Affiliations:** ^1^ Renal Department and Nephrology Institute, Sichuan Provincial People's Hospital, School of Medicine, University of Electronic Science and Technology of China, Chengdu 610072, China

**Keywords:** IgA nephropathy, CARD9, VAV3, single nucleotide polymorphism, case-control study

## Abstract

**Background:**

Immunoglobulin A nephropathy (IgAN), the most common form of primary glomerular diseases worldwide, is a complex multifactorial disease. Previous genome wide association studies (GWAS) reported that variants CARD9 and VAV3 genes were associated with immunoregulation and susceptibility to IgAN. In this study, we further validated the associations and explored the interaction effect of rs4077515 and rs17019602 in IgAN patients.

**Results:**

There was no significant correlation between the two variants and IgAN (*P* > 0.05). The gene-gene analysis showed that rs4077515 and rs17019602 had interaction effect on the susceptibility to IgAN. For additive interaction, the CT or TT of rs4077515 and GG of 17019602 genotype combination conferred a 2.56-fold risk of IgAN reference to CC of 4077515 and AA of 17019602 (OR = 2.56, 95% CI: 0.98–6.69, *P* = 0.049). In our study, clinical data was available for 543 patients. In comparison, neither rs4077515 nor rs17019602 showed significant association between genotype distribution and clinical parameters in IgAN patients (*P* > 0.05).

**Materials and Methods:**

The case-control study included 586 patients with IgAN and 606 healthy controls. Variant rs4077515 of CARD9 gene and rs17019602 of VAV3 gene were genotyped by the ABI TaqMan probe assay.

**Conclusions:**

The interaction effect of the variants of CARD9 and VAV3 genes increases the susceptibility to IgAN.

## INTRODUCTION

Immunoglobulin A nephropathy (IgAN), one of the common causes resulting the end-stage renal disease (ESRD), is the most common form of primary glomerular diseases worldwide [1, 2]. Diagnosis of IgAN depends on kidney biopsy. Now IgAN is considered to be a kind of autoimmune-mediated inflammatory disease involving both adaptive and innate immunity, but the pathogenesis is still poorly understood [3].

The development and progression of IgAN were result of multiple factors, including environment and genetic. Familial aggregation, inter-individual variation, as well as differences among different ethnics and geographical distributions all supported that genetic factor gave clues to the pathophysiology of IgAN [4–6]. The recently published four large-scale genome-wide association studies (GWAS) had identified lots of genetic risk loci which can explain 6–8% of total disease risk [5–8]. These GWAS studies were not only identified many susceptible loci but also highlighted several pathogenetic pathways of IgAN, for example, variants in major histocompatibility complex (MHC) region, CFHR1/3, TNFSF13, and DEFA respectively implicated pathways of antigen processing and presentation, the complement system, regulation of mucosal IgA production and innate immunity [5–8]. It was very interesting that two new single nucleotide polymorphisms (SNPs), rs4077515 in CARD9 gene and rs17019602 in VAV3 gene, were previously reported to associate with immunity against intestinal pathogens [7]. Host-intestinal pathogen interactions acted on the genetic landscape of IgAN. The genetic risk correlating with variation in local pathogens and the multi-locus adaptation led by geospatial distribution of risk alleles provided tangible evidence. In addition, rs4077515 and rs17019602 were associated with inflammatory bowel disease (IBD), maintaining the intestinal epithelial barrier and resulting in mucosal pathogens [7].

Recently, several GWAS have reported that variants of CARD9 gene were associated with immunity diseases, including Crohn's disease (CD), [9] ulcerative colitis (UC), [9, 10] primary sclerosing cholangitis (PSC), [10] and Candida infections [11]. Besides, variants of VAV3 gene related to Hashimoto thyroiditis (HT) [12] and UC [13]. It is very interesting that the variants of CARD9 and VAV3 genes were also shown to associate with susceptibility to IgAN by the GWAS study mentioned above, [7] which was directly responsible for IgAN. In this study, we further validated the associations and explored the interaction effect of rs4077515 in CARD9 gene and rs17019602 in VAV3 gene in Chinese Han IgAN population.

## RESULTS

### Comparisons of CARD9 and VAV3 SNPs between IgAN patients and healthy controls

The genotype and allele frequency distributions of rs4077515 and rs17019602 in healthy controls and patients were showed in Table 1 and Table 2, respectively. There were no deviations from Hardy-Weinberg equilibrium for rs4077515 and rs17019602 in the case groups (*P* = 0.70 and 0.78, respectively) and control groups (*P* = 0.53 and 0.11, respectively). For rs4077515, T allele was the minor allele and the frequency in case group was higher than in control group, but the difference was not significant (*P* = 0.160). The frequencies of rs4077515 genotypes CC, CT, and TT were not significantly different between the IgAN patients and the healthy controls (*P* = 0.287) (Table 1). For rs17019602, the allele frequency distributions in case and control groups were not significantly different (*P* = 0.630). The frequencies of AA, AG, and GG genotypes in the case group were 61.4%, 34.2%, 4.4%, respectively, and in the control group were 61.8%, 35.0%, 3.2%, respectively. But significant differences were not observed between case and control groups (*P* = 0.541) (Table 2).

**Table 1:** The associations between variant rs4077515 of CARD9 gene and IgAN

Rs4077515	Genotype	Control (%)	case (%)	*P*-Value	Log-*P* value	ORs
Total		595	586			
Codominant	C/C	294 (49.4)	273 (46.6)	0.287	0.159	1.134(0.952–1.352)
	C/T	253 (42.5)	251 (42.8)			
	T/T	48 (8.1)	62 (10.6)			
Dominant	C/C	294 (49.4)	273 (46.6)	0.331	0.331	1.120(0.891–1.407)
	C/T-T/T	301 (50.6)	313 (53.4)			
Recessive	C/C-C/T	547 (91.9)	524 (89.4)	0.137	0.138	1.348(0.908–2.002)
	T/T	48 (8.1)	62 (10.6)			
Allele	C allele	841 (70.7)	797 (68.0)	0.160	0.160	1.134(0.952–1.351)
	T allele	349 (29.3)	375 (32.0)			

*P*-value was calculated for chi-square test.

Log-*P* value was calculated for logistic regression.

**Table 2:** The associations between variant rs17019602 of VAV3 gene and IgAN

Rs17019602	Genotype	Control (%)	case (%)	*P*-Value	Log-*P* value	ORs
Total		591	585			
Codominant	A/A	365 (61.8)	359 (61.4)	0.541	0.623	1.052(0.860–1.287)
	A/G	207 (35.0)	200 (34.2)			
	G/G	19 (3.2)	26 (4.4)			
Dominant	A/A	365 (61.8)	359 (61.4)	0.890	0.890	1.017 (0.804–1.286)
	A/G-G/G	226 (38.2)	226 (38.6)			
Recessive	A/A-A/G	572 (96.8)	559 (95.6)	0.272	0.219	1.454(0.800–2.643)
	G/G	19 (3.2)	26 (4.4)			
Allele	A allele	937 (79.3)	918 (78.5)	0.630	0.630	1.050(0.861–1.28)
	G allele	245 (20.7)	252 (21.5)			

*P*-value was calculated for chi-square test.

Log-*P* value was calculated for logistic regression.

We further explore the association of these two SNPs with IgAN using two different genetic models which were dominant and recessive models. Compared with the control group, though the OR values of recessive model both in rs4077515 (OR = 1.348, 95% CI: 0.908–2.002, *P* = 0.137) and rs17019602 (OR = 1.400, 95% CI: 0.766–2.559, *P* = 0.272) showed an increased risk trend of IgAN, no statistical correlations were identified between the two models and IgAN (Tables 1 and 2).

### The interaction effect of rs4077515 and rs17019602 was associated with IgAN

**Figure 1:**
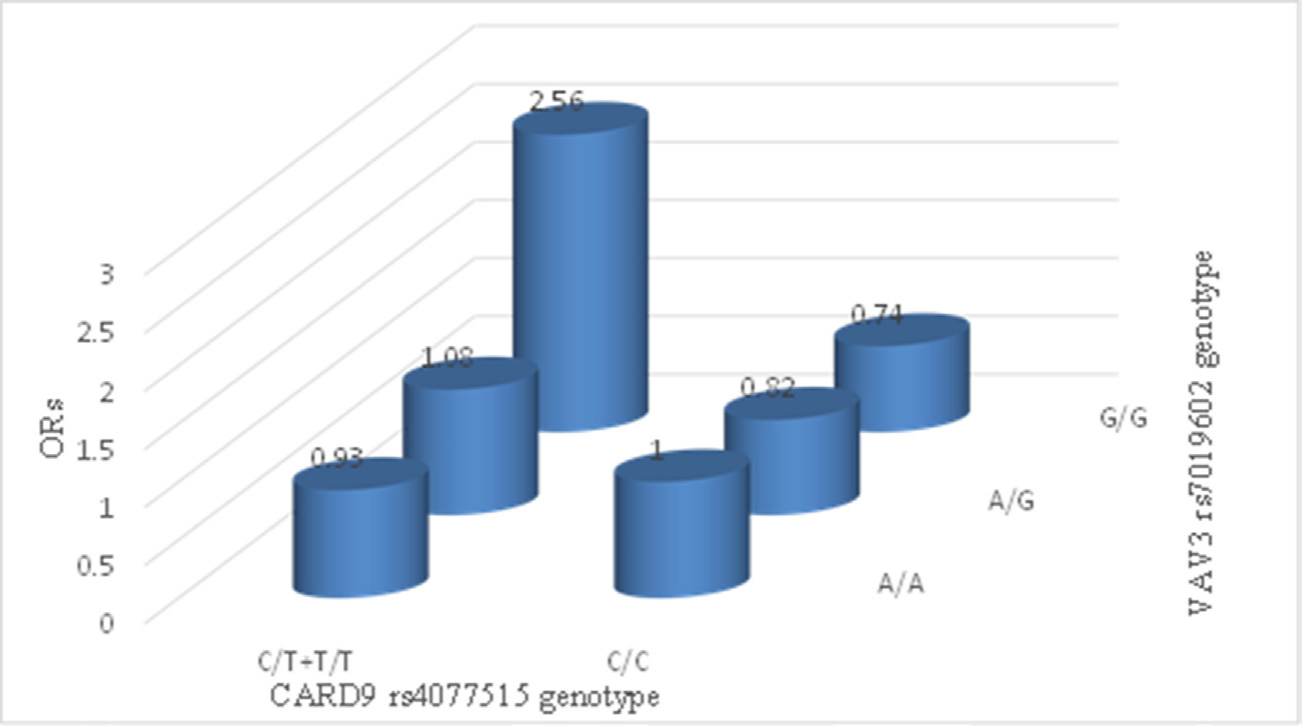
The analysis of gene-gene interaction X-axis and Z-axis standed for the genotypes of rs4077515 and rs17019602, respectively, and the Y-axis standed for the OR value. CC/TT genotype combination was set as the reference. The combination of risk genotypes of rs4077515 and rs17019602, CT or TT and GG, conferred a 2.56-fold higher risk for the susceptibility to IgAN compared to the reference (OR = 2.56, 95% CI: 0.98–6.69, *P* = 0.049). Other ORs were not significant.

To explore the potential SNP-SNP interaction effect between IgAN patients and healthy controls, we conducted the gene-gene analysis which performed between rs4077515 and rs17019602. This analysis was performed under the assumption that T-allele of rs4077515 and G-allele of rs17019602, the minor alleles, were the risk alleles. According to the number of the risk allele about both rs4077515 and rs17019602, 0–5 were assigned to patients with IgAN and healthy controls, showing CC/AA, CT or TT/AA, CC/AG, CT or TT/AG, CC/GG, CT or TT/GG, respectively. In this study, CC/TT genotype combination was set as the reference and a positive correlation was found between case and control groups. For interaction, the combination of risk genotypes of rs4077515 and rs17019602, CT or TT and GG, conferred a 2.56-fold higher risk for the susceptibility to IgAN compared to the reference (OR = 2.56, 95% CI: 0.98–6.69, *P* = 0.049) (Figure 1), indicating that rs4077515 and rs17019602 had interaction effect on the susceptibility to IgAN.

### Comparisons of clinical characteristics among patients with different genotypes of rs4077515 and rs17019602

In our cohort, complete clinical data was available for 543 patients. To explore the association with genotype distribution and clinical characteristics in IgAN patients, we analyzed the difference in the genotype distributions according to clinical characteristics such as SCr, ALB, eGFR, 24-hour urine protein (Table 3). In comparison, neither rs4077515 nor rs17019602 showed significant difference in the clinical characteristics (*P* > 0.05).

**Table 3:** Comparisons of clinical characteristics among patients with different genotypes of rs4077515 and rs17019602

characteristics	CARD9					VAV3				
	C/C	C/T	T/T	*P*-value	χ^2^	A/A	A/G	G/G	*P*-value	χ^2^
Male/female	101/149	96/139	21/37	0.807 ^a^		132/200	75/111	10/14	0.978 ^a^	
Age(year)	34.50 ± 12.2	34.01 ± 11.41	32.31 ± 9.91	0.787	0.478	33.44 ± 11.44	34.92 ± 12.22	35.2 ± 9.52	0.447	1.612
SCr (μmol/L)	104.18 ± 84.56	100.89 ± 45.02	97.92 ± 34.81	0.237	2.882	105.65 ± 76.73	94.53 ± 42.10	111.41 ± 47.41	0.252	2.754
ALB (g/L)	39.40 ± 9.73	39.00 ± 7.60	38.61 ± 8.32	0.911	0.186	39.66 ± 8.94	38.36 ± 8.31	37.85 ± 8.16	0.122	4.203
Serum IgA (g/L)	3.26 ± 2.47	2.98 ± 2.29	3.11 ± 2.62	0.147	3.832	3.19 ± 2.55	3.01 ± 2.20	3.18 ± 2.28	0.905	0.199
Serum C3(g/L)	0.98 ± 0.29	1.03 ± 0.26	0.98 ± 0.23	0.301	2.398	1.03 ± 0.28	0.96 ± 0.29	0.98 ± 0.16	0.217	3.054
Urine protein (g/24h)	2.00 ± 2.08	1.84 ± 1.84	2.24 ± 2.19	0.481	1.465	1.94 ± 1.99	1.99 ± 2.02	2.06 ± 2.03	0.968	0.066
eGFR (ml/(min·1.73m^2^))	87.32 ± 38.38	83.26 ± 34.77	82.34 ± 30.48	0.323	2.261	84.20 ± 34.50	88.03 ± 38.67	74.59 ± 34.62	0.248	2.793
HGB(g/L)	131.28 ± 17.87	130.64 ± 21.11	138.83 ± 17.21	0.115	4.323	132.95 ± 20.37	130.37 ± 17.19	124.40 ± 16.71	0.208	3.139
UA(umol/L)	370.72 ± 117.83	370.05 ± 114.60	348.73 ± 114.79	0.669	0.805	367.83 ± 119.46	365.15 ± 112.57	386.78 ± 98.02	0.508	1.353
WBC(10^9^/L)	7.05 ± 2.42	7.25 ± 2.92	7.03 ± 2.38	0.909	0.191	7.23 ± 2.28	6.94 ± 3.05	7.41 ± 2.84	0.130	4.081
LYM(10^9^/L)	1.71 ± 0.73	1.77 ± 0.70	1.67 ± 0.71	0.436	1.662	1.77 ± 0.70	1.68 ± 0.76	1.63 ± 0.50	0.226	2.973

*P*-value and χ^2^ were calculated for Kruskal-Wallis H test.

^a^ This *P*-value was calculated for chi-square test.

## DISCUSSION

We conducted a case-control association study of the variants of CARD9 and VAV3 genes in IgAN. At first, the association analysis between rs4077515 or rs17019602 and IgAN demonstrated that there was no significant correlation between single SNP and IgAN. As a 2-locus combination, the analysis of disease risk score showed that rs4077515 and rs17019602 had interaction effect on the susceptibility to IgAN. The combination of risk genotypes of rs4077515 and rs17019602, CT or TT and GG, conferred a 2.56-fold higher risk for the susceptibility to IgAN while compared with the reference. Otherwise, there was no significant association between genotype distribution and clinical characteristics in IgAN patients.

CARD9 is a nonredundant adapter protein which functions in the immune system. As an adaptor protein, CARD9 can coordinate innate and adaptive immune responses, mounting an IFN-γ gut response to pathogens appropriately [15] and stimulating T cells to differentiate into TH17 cells [16]. Previous researches have demonstrated that CARD9 was associated with some inflammatory diseases such as tuberculosis, [17] IBD, [18] CD [9] and rheumatoid arthritis [19]. Besides, as the family of guanine nucleotide exchange factors (GEFs), VAV proteins, including VAV1, VAV2, and VAV3, activate Rho guanosine triphosphatases (GTPases). VAV3, which is crucial for B and T lymphocyte development, is widely expressed compared with VAV1 that is specifically expressed in lymphoid lineage cells [20]. In the process of autoimmune β-cell destruction, alteration of VAV3 expression is one of the etiological factors [21].

Kiryluk had first reported that several new risk loci, including variants CARD9 and VAV3 genes, were associated with immunoregulation and the susceptibility to IgAN, which involved in the multi-hit pathogenesis of IgAN [7]. CARD9 was a proinflammatory which regulated intestinal inflammation and production of IgA, potentially modulating Hit1 [7, 22]. VAV3 was important for antigen presentation, which potentially modulated Hit1 through regulating IgA production and modulated Hit4 through glomerular inflammation, clearance of immune complexes and phagocytosis [7, 22]. These results demonstrated that IgAN and other immune-mediated diseases share some risk loci. Taken the results of this study and our study together, it further supported that IgAN was an immune-mediated disease which involved in inflammation and mucosal immunity. Macroscopic hematuria was often concurrent with an infection and may subsequently aggravated the disease [23]. IgAN was the most frequent kidney biopsy diagnosis in IBD [24]. Those previous studies were consistent with our results.

Furthermore, CARD9 which encoded a molecular scaffold for the assembly of a BCL10 signaling complex that activates NF-κB belonged to NOD-like receptor signaling pathway. At the same time, VAV3 was also important for NF-κB activation in B cells. However, the detail mechanism of CARD9 and VAV3 SNPs involving the pathogenesis of IgAN remains to be further studied.

In conclusion, CARD9 and VAV3 were both related in the inflammation and mucosal immunity, sharing the similar mechanism to IgAN, which provided the basis of our result that the variants of CARD9 and VAV3 genes had interaction effect which increased the susceptibility to IgAN.

To our knowledge, this is the first study regarding the relationships and interaction effect of the variants of CARD9 and VAV3 genes in IgAN populations from Southwest China. But our study had several limitations. First, we didn't have the follow-up data to assess the relationships between the variants of the two genes and the outcomes of IgAN patients. Second, the relatively small sample size of our cohort may not result the enough statistical power to reveal the correlation between single SNP and the susceptibility to IgAN. Besides, for interaction, the reason why the combination of the CT or TT of rs4077515 and AA of rs17019602 just conferred 0.93-fold risk of reference maybe also be resulted in the small sample. The further large sample size study for exploring the relationship is needed.

## MATERIALS AND METHODS

### Study population and clinical data

All patients with IgAN and the healthy controls were unrelated Han Chinese individuals recruited from Southwest China. All the cases and controls were collected from a general hospital, which covered more than 80 million people in Southwest China. All patients underwent the renal biopsy and were histopathologically diagnosed according to the criteria of the World Health Organization. A total of 586 patients were enrolled in this study and any of the patients was excluded as followings: 1) Systemic diseases, for example systemic lupus erythematosus (SLE), diabetes and chronic liver disease. 2) Pregnant women. 3) Patients with renal transplantation. Total 606 healthy individuals without diabetes, hypertension and acute or chronic kidney diseases served as the healthy controls. Clinical data at the time of renal biopsy, such as blood pressure, serum creatinine and 24-hour urine protein, were collected from all patients. The estimated glomerular filtration rate (eGFR) was calculated by the CKD-EPI equation [14]. The protocol for study was approved by the Ethics Committee of Sichuan Provincial People's Hospital, and informed consent was obtained from every participant.

### DNA extraction and genotyping

The genomic DNA was extracted from peripheral bloods which were collected from all patients with IgAN and the healthy controls using the RelaxGene Blood DNA System Kit (Tiangen, Beijing city, China) according to the manufacturer's protocol. The genotyping was conducted by real-time PCR, performed using an ABI 7500 Fast Real-time PCR System (ABI, Foster city, CA). Sequences of SNPs were found in the previous report [7] and TaqMan^®^ probe assays were obtained from Applied Biosystems(ABI, Foster city, CA), as well as the genotyping master mix. Assays were run at the final volume of 10 ul, which consisted of 5 ul of TaqMan^®^Gene Expression Master mix, 0.25 ul of TaqMan^®^ SNP Genotyping Assay, 2.75 ul of Nuclease-free water and 2ul target DNA. In the plate document in the case of real-time PCR systems, select the Standard mode thermal cycling setting.

In our cohort, 586 patients with biopsy proven IgAN and 606 healthy controls were included, in which rs4077515 were successfully genotyped in 586 cases and 595 controls, and rs17019602 in 585 cases and 591 controls. 11 healthy individuals were not successful because of poor quality of DNA.

### Statistical analyses

All data analyses were performed using SPSS 21.0 software version (SPSS, Inc, Chicago, IL). Hardy-Weinberg equilibrium (HWE) of SNPs was tested in cases and healthy controls using a standard Chi-square test. The Kruskal–Wallis H test was used when the data was not a normal distribution which the clinical characteristics belonged to. Frequency distributions of the genotypes and alleles in the case and control groups and the additive interaction effect were analyzed using the Chi-squared test and the binary logistic regression analysis adjusted for gender and age. The strength was indicated by *P*-value and an odds ratio (OR) with a 95% confidence interval (CI). *P*-value < 0.05 was considered statistically significant.

## CONCLUSIONS

In summary, our result indicated that the interaction effect of variants CARD9 and VAV3 genes increased the susceptibility to IgAN. There was no significant correlation between the two variants and clinical characteristics of IgAN patients.

## References

[1] Wyatt RJ, Julian BA (2013). IgA The New Engnephropathy. N Engl J Med.

[2] D'Amico G (1987). The cThe New Engommonest glomerulonephritis in the world: IgA nephropathy. Q J Med.

[3] Kiryluk K, Novak J, Gharavi AG (2013). Pathogenesis of immunoglobulin A nephropathy: recent insight from genetic studies. Annu Rev Med.

[4] Julian BA, Quiggins PA, Thompson JS, Woodford SY, Gleason K, Wyatt RJ (1985). Familial IgA nephropathy. Evidence of an inherited mechanism of disease. N Engl J Med.

[5] Gharavi AG, Kiryluk K, Choi M, Li Y, Hou P, Xie J, Sanna-Cherchi S, Men CJ, Julian BA, Wyatt RJ, Novak J, He JC, Wang H (2011). Genome-wide association study identifies susceptibility loci for IgA nephropathy. Nat Genet.

[6] Yu XQ, Li M, Zhang H, Low HQ, Wei X, Wang JQ, Sun LD, Sim KS, Li Y, Foo JN, Wang W, Li ZJ, Yin XY (2011). A genome-wide association study in Han Chinese identifies multiple susceptibility loci for IgA nephropathy. Nat Genet.

[7] Kiryluk K, Li Y (2014). Discovery of new risk loci for IgA nephropathy implicates genes involved in immunity against intestinal pathogens. Nat Genet.

[8] Feehally J, Farrall M, Boland A, Gale DP, Gut I, Heath S, Kumar A, Peden JF, Maxwell PH, Morris DL, Padmanabhan S, Vyse TJ, Zawadzka A (2010). HLA has strongest association with IgA nephropathy in genome-wide analysis. J Am Soc Nephrol.

[9] Zhernakova A, Festen EM, Franke L, Trynka G, van Diemen CC, Monsuur AJ, Bevova M, Nijmeijer RM, van 't Slot R, Heijmans R, Boezen HM, van Heel DA, van Bodegraven AA (2008). Genetic analysis of innate immunity in Crohn's disease and ulcerative colitis identifies two susceptibility loci harboring CARD9 and IL18RAP. Am J Hum Genet.

[10] Janse M, Lamberts LE, Franke L, Raychaudhuri S, Ellinghaus E, Muri Boberg K, Melum E, Folseraas T, Schrumpf E, Bergquist A, Bjornsson E, Fu J, Jan Westra H (2011). Three ulcerative colitis susceptibility loci are associated with primary sclerosing cholangitis and indicate a role for IL2, REL, and CARD9. Hepatology.

[11] Smeekens SP, van de Veerdonk FL, Kullberg BJ, Netea MG (2013). Genetic susceptibility to Candida infections. EMBO Mol Med.

[12] Oryoji D, Ueda S, Yamamoto K, Yoshimura Noh J, Okamura K, Noda M, Watanabe N, Yoshihara A, Ito K, Sasazuki T (2015). Identification of a Hashimoto thyroiditis susceptibility locus via a genome-wide comparison with Graves' disease. J Clin Endocrinol Metab.

[13] Jones CI, Bray S, Garner SF, Stephens J, de Bono B, Angenent WG, Bentley D, Burns P, Coffey A, Deloukas P, Earthrowl M, Farndale RW, Hoylaerts MF (2009). A functional genomics approach reveals novel quantitative trait loci associated with platelet signaling pathways. Blood.

[14] Hebert SA, Molony DA (2015). ACP Journal Club: the CKD-EPI equation for eGFR predicted adverse outcomes after PCI better than other equations. Ann Intern Med.

[15] Fairfax BP, Humburg P, Makino S, Naranbhai V, Wong D, Lau E, Jostins L, Plant K, Andrews R, McGee C, Knight JC (2014). Innate immune activity conditions the effect of regulatory variants upon monocyte gene expression. Science.

[16] Burghardt KM, Avinashi V, Kosar C, Xu W, Wales PW, Avitzur Y, Muise A (2014). A CARD9 polymorphism is associated with decreased likelihood of persistent conjugated hyperbilirubinemia in intestinal failure. PloS One.

[17] Bowker N, Salie M, Schurz H, van Helden PD, Kinnear CJ, Hoal EG, Moller M (2016). Polymorphisms in the Pattern Recognition Receptor Mincle Gene (CLEC4E) and Association with Tuberculosis. Lung.

[18] Wang Z, Fan R, Wang L, Zhou J, Zheng S, Hu S, Chen M, Zhang T, Lin Y, Zhang M, Zhong J (2015). Genetic association between CARD9 variants and inflammatory bowel disease was not replicated in a Chinese Han population. Int J Clin Exp Pathol.

[19] Arya R, Del Rincon I, Farook VS, Restrepo JF, Winnier DA, Fourcaudot MJ, Battafarano DF, de Almeida M, Kumar S, Curran JE, Jenkinson CP, Blangero J, Duggirala R (2015). Genetic Variants Influencing Joint Damage in Mexican Americans and European Americans With Rheumatoid Arthritis. Genet Epidemiol.

[20] Fujikawa K, Iwata T, Inoue K, Akahori M, Kadotani H, Fukaya M, Watanabe M, Chang Q, Barnett EM, Swat W (2010). VAV2 and VAV3 as candidate disease genes for spontaneous glaucoma in mice and humans. PloS One.

[21] Fraser HI, Dendrou CA, Healy B, Rainbow DB, Howlett S, Smink LJ, Gregory S, Steward CA, Todd JA, Peterson LB, Wicker LS (2010). Nonobese diabetic congenic strain analysis of autoimmune diabetes reveals genetic complexity of the Idd18 locus and identifies Vav3 as a candidate gene. J Immunol (Baltimore, Md: 1950).

[22] Magistroni R, D'Agati VD, Appel GB, Kiryluk K (2015). New developments in the genetics, pathogenesis, and therapy of IgA nephropathy. Kidney Int.

[23] Mestecky J, Raska M, Julian BA, Gharavi AG, Renfrow MB, Moldoveanu Z, Novak L, Matousovic K, Novak J (2013). IgA nephropathy: molecular mechanisms of the disease. Annu Rev Pathol.

[24] Ambruzs JM, Walker PD, Larsen CP (2014). The histopathologic spectrum of kidney biopsies in patients with inflammatory bowel disease. Clin J Am Soc Nephrol.

